# Combination treatment including targeted therapy for advanced hepatocellular carcinoma

**DOI:** 10.18632/oncotarget.11954

**Published:** 2016-09-10

**Authors:** Jianzhen Lin, Liangcai Wu, Xue Bai, Yuan Xie, Anqiang Wang, Haohai Zhang, Xiaobo Yang, Xueshuai Wan, Xin Lu, Xinting Sang, Haitao Zhao

**Affiliations:** ^1^ Department of Liver Surgery, Peking Union Medical College Hospital, Chinese Academy of Medical Sciences and Peking Union Medical College (CAMS & PUMC), Beijing, China; ^2^ Center of Translational Medicine, Peking Union Medical College Hospital, Chinese Academy of Medical Sciences and Peking Union Medical College, Beijing, China

**Keywords:** targeted therapy, combination treatment, hepatocellular carcinoma, molecular targeted agents

## Abstract

Management of advanced hepatocellular carcinoma (HCC), one of the most lethal cancers worldwide, has presented a therapeutic challenge over past decades. Most patients with advanced HCC and a low possibility of surgical resection have limited treatment options and no alternative but to accept local or palliative treatment. In the new era of cancer therapy, increasing numbers of molecular targeted agents (MTAs) have been applied in the treatment of advanced HCC. However, mono-targeted therapy has shown disappointing outcomes in disease control, primarily because of tumor heterogeneity and complex cell signal transduction. Because incapacitation of a single target is insufficient for cancer suppression, combination treatment for targeted therapy has been proposed and experimentally tested in several clinical trials. In this article, we review research studies aimed to enhance the efficacy of targeted therapy for HCC through combination strategies. Combination treatments involving targeted therapy for advanced HCC are compared and discussed.

## INTRODUCTION

HCC is the fifth most commonly diagnosed cancer and the second most common cause of cancer deaths worldwide [1]. For most patients with HCC, the diagnosis is delayed and the prognosis is poor. The median overall survival (OS) in patients with advanced HCC is less than 1 year, mainly owing to the absence of effective therapies [2]. When the diagnosis is confirmed, 70% to 80% of HCC patients have lost the opportunity to undergo complete tumor resection [3]. As it is impossible to perform curative therapies, patients with advanced HCC have to rely on non-surgical therapies such as chemotherapy, transcatheter arterial chemoembolization or embolization (TACE or TAE), radiotherapy, percutaneous ethanol injection (PEI), targeted therapy, or immunotherapy to prolong their survival time [4–7].

Although there are various options for non-surgical management of HCC, few therapies appear to effectively improve prognosis [8, 9]. In the new era of the war against cancer, targeted therapy based on MTAs has gradually became an indispensable component of the treatment regimen for patients with advanced cancer. However, in many cases, low efficacy and drug resistance hamper the clinical application of MTAs, especially in the treatment of HCC [10]. Most MTAs, such as sunitinib, brivanib, linifanib, everolimus, ramucirumab, and sorafenib, show only a slight anticancer effect in advanced HCC [11]. For example, sorafenib, a small inhibitor of multiple tyrosine protein kinases that is considered the most efficient targeted drug for advanced HCC to date, has been proved to extend median OS by only 3 months with tolerable adverse events according to two large sample clinical trials [12, 13]. Moreover, patients who initially respond to therapy eventually suffer cancer progression. The median progression-free survival (PFS) is prolonged by MTAs, but few patients achieve complete response and extended OS [14].

To counteract the low efficacy of monotargeted therapy, the concept of combination therapy based on MTAs for the comprehensive treatment of cancer has been proposed in recent years [15], including in the treatment of HCC [16]. Here, we reviewed relevant studies and clinical trials concerning combination treatments based on targeted therapy agents for advanced HCC, and discuss the current status of clinical applications for this novel strategy.

## TARGETED THERAPY COMBINED WITH CHEMOTHERAPY

Chemotherapy, the use of chemical agents for the treatment of cancer, is generally the adjuvant of choice for metastatic disease when alternative treatment options are limited [17]. A typical chemotherapeutic scheme includes several drugs that possess diverse anticancer mechanisms, such as topoisomerase inhibitors, cytotoxic antibiotics, or spindle poisons. The main aim of combining different agents is to counteract the aberrant molecular events that promote carcinogenesis and tumor progression [18]. However, chemotherapy is impotent in HCC, and most chemotherapeutic agents used alone or in combination are ineffective and relatively toxic to patients [19]. The low efficacy of traditional cytotoxic chemotherapy for managing advanced HCC was previously considered a therapeutic challenge. It is anticipated that enhanced antitumor effects can be achieved through combined application of MTAs and chemotherapy. Furthermore, the doses of MTAs or chemotherapeutic agents can be reduced when given in combination, which might alleviate the adverse effects (AEs) of the drugs [20]. In recent years, several hypotheses related to chemotherapy combined with targeted therapy have been tested in advanced HCC, mainly concentrating on chemotherapy combined with receptor tyrosine kinase inhibitors (RTKIs) or anti-angiogenesis clonal antibodies (Table 1).

**Table 1:** Clinical trials regarding chemotherapy combine with targeted therapy (with published results)

Agents	Stage	Patients(n)	Therapeutic shceme	First or second line	Efficacy (combined therapy *vs* monotherapy)	Adverse events (AEs)	Ref
Doxorubici + Sorafenib	Phase 2	96	Sorafenib 400mg bid po plus doxorubicin 60mg/m^2^/21days i.v. *n* = 47) *vs* doxorubicin monotherapy (60mg/m^2^/21 days i.v. *n* = 48)	First line	mTTP: 6.4 *vs*. 2.8 months mPFS: 6.0 *vs*. 2.7 months mOS: 13.7 *vs*. 6.5 months	fatigue, dermatology/skin, hand-foot skin reaction, hematologic (neutropenia, leukopenia)	[1]
Erlotinib + Docetaxel	Phase 1	25 (14 HCC)	Docetaxel 30mg/m^2^ i.v. plus erlotinib 150 mg po of each 28-day cycle.	First line	16-week PFS: 38%-HCC. mOS: 6.7 months-HCC	rash, diarrhea, fatigue	[2]
Bevacizumab + Capecitabine	Phase 2	45	Bevacizumab 7.5 mg/kg i.v. plus capecitabine 800 mg/m^2^ bid po every 3 weeks.	First line	ORR: 9%; DCR: 52% mPFS: 2.7 months mOS: 5.9 months	diarrhoea, nausea/vomiting, gastrointestinal bleeding, hand–foot syndrome, lower respiratory tract infection and proteinuria.	[3]
Bevacizumab+ Capecitabine + Oxaliplatin	Phase 2	40	Each treatment cycle was 21 days. Bevacizumab 5 mg/kg i.v. and oxaliplatin 130 mg/m^2^ i.v. Capecitabine 825 mg/m^2^ bid po.	First line	mPFS: 6.8 months mOS: 9.8 months DCR: 77.5%	sensory neuropathy, fatigue and diarrhea.	[4]
Gemcitabin + Oxaliplatin + Bevacizumab	Phase 2	30	For cycle 1 (14 days), bevacizumab 10 mg/kg alone i.v. For cycle 2 and beyond (28 days/cycle), bevacizumab 10 mg/kg. Gemcitabine 1,000 mg/m^2^. Oxaliplatin at 85 mg/m^2^.	First line	ORR: 20% mOS: 9.6 months mPFS: 5.3 months.	leukopenia/neutropenia, transient elevation of aminotransferases, hypertension and fatigue.	[5]
Gemcitabine + Oxaliplatin (GEMOX) + Cetuximab	Phase 2	45	Cetuximab 400 mg/m^2^ initially then 250 mg/m^2^ weekly; gemcitabine 1000 mg/m^2^; oxaliplatin 100 mg/m^2^.	First line	mPFS: 4.7 months mOS: 9.5 months 1-year survival rate: 40%.	thrombocytopenia, neutropenia and anemia.	[6]
Capecitabine + Oxaliplatin + Cetuximab	Phase 2	29	oxaliplatin 130 mg/m^2^ i.v. plus cetuximab 400 mg/m^2^ IV on day 1 of cycle 1 followed by 250 mg/m^2^ iv weekly, capecitabine 850 mg/m^2^PO Bid.	First line	DCR: 83% mTTP: 4.5 months mPFS: 3.3 months mOS: 4.4 months.	fatigue, diarrhea, and mucositis.	[7]

**Abbreviations:** mOS: median overall survival; mPFS: median progression-free survival; mTTP: median time to progression; DCR: disease control rate; ORR: objective response rate; PFR: progression-free rate.

### RTKIs plus chemotherapy

Receptor tyrosine kinases (RTKs) are the high-affinity cell surface receptors for many polypeptide growth factors, cytokines and hormones and are essential for cell signal transduction of normal cells and cancer cells [21]. Targeted agents that inhibit RTKs account for a large percentage of the MTAs used in HCC. To date, the therapeutic strategy involving RTKIs and chemotherapy to treat HCC is confined to the use of sorafenib or erlotinib plus cytotoxic agents. The most promising results have been presented for sorafenib plus doxorubicin [22]. Doxorubicin, which functions by intercalating into the DNA, has a definite effect in repressing the progression of HCC, but this is accompanied by various AEs. Among these AEs, the most serious is life-threatening heart damage [23]. When doxorubicin is combined with sorafenib, patients may receive additional clinical benefits. Abou-Alfa et al. evaluated the efficacy and safety of combination treatment with doxorubicin and sorafenib [22]. In this double-blind controlled phase II study, 96 patients with advanced HCC were randomly assigned to receive doxorubicin (60 mg/m^2^) plus sorafenib or placebo (400 mg twice daily). The outcome from this trial was promising, and showed significant prolongation of median time to progression (TTP), OS, and PFS compared with the control arm (doxorubicin plus placebo). Notwithstanding the absence of a comparative sorafenib standard group in the research, this trial illustrates that sorafenib plus doxorubicin may contribute to treating advanced HCC. In addition, relevant clinical research is ongoing in a phase III trial (NCT01015833) with a larger sample size and a sorafenib-controlled arm. This therapeutic setting is rational because anthracycline antibiotics such as doxorubicin have been demonstrated to inhibit angiogenesis [24] and thus may perform complementary inhibitory functions when administered in combination with sorafenib. Moreover, suppression of the RAS/RAF/MEK/ERK pathway can partly alleviate drug resistance in malignant tumors [25]. Others combinations (Table 1) involving erlotinib plus docetaxel or erlotinib plus gemcitabine and oxaliplatin show insignificant antitumor outcomes in HCC and biliary cancer [26]. Intriguingly, the subset of patients with negative/low E-cadherin expression or K-Ras mutation might gain valid tumor control. Considering that the specific target of erlotinib is the epidermal growth factor receptor (EGFR) [27], this phenomenon hints that increased clinical benefits of RTKIs plus chemotherapy might be achieved through precise selection of eligible patients.

### Anti-angiogenesis clonal antibodies plus chemotherapy

Clonal antibodies targeting angiogenesis ligands or receptors have been widely applied to treat various kinds of cancer [28]. Because vascular growth is one of the essential factors in the occurrence and development of HCC, the role of anti-angiogenesis agents in HCC therapy is quite significant [29]. However, anti-angiogenesis antibodies alone often show an inadequate anticancer effect in the treatment of patients with advanced HCC [30]. For instance, use of bevacizumab, a humanized recombinant monoclonal antibody that binds all isoforms of circulating VEGF-A, has been validated in many solid tumors. Monotherapy using bevacizumab in patients with advanced HCC, however, merely achieves a partial response [31]. When bevacizumab is combined with chemotherapeutic agents, it may strengthen chemotherapy activity and enhance chemosensitivity [32, 33] despite its direct antiangiogenic effects. The combination of bevacizumab with chemotherapy has been shown to provide clinical benefits in patients with breast, lung, and colorectal cancer and has been tested in patients with HCC [34]. For example, the GEMOX-B regimen containing gemcitabine, oxaliplatin, and bevacizumab demonstrated moderate antitumor activity and good tolerability in patients with unresectable or metastatic HCC [35]. Other regimens of combination therapy, such as capecitabine plus bevacizumab with or without oxaliplatin, have also been tested with outcomes similar to those of GEMOX-B [36, 37]. Moreover, metronomic capecitabine was presented as a potential second-line treatment for HCC after development of sorafenib resistance in a retrospective study. The disease control rate (DCR) was 23% and median OS reached 8 months in this study [38]. These results demonstrate that combined application of bevacizumab and chemotherapeutic agents could be well tolerated and may open up alternative therapeutic options for treating advanced HCC.

Another antitumor clonal antibody, cetuximab, a monoclonal antibody with anticancer biological activity through inhibition of EGFR, has been demonstrated to possess enhanced effectiveness in combination with chemotherapy in both colorectal cancer and HCC [39, 40]. Several lines of evidence have proved that blockade of the EGFR pathway can increase the response rate of radiotherapy or chemotherapy in EGFR-activated carcinomas [41]. Therefore, potent suppression of angiogenesis by combination treatment with chemotherapy and targeted therapy may be an attractive option for patients with advanced HCC.

## CO-INHIBITION OF DUAL OR MULTIPLE TARGETS

Although many MTAs have been used to treat HCC, most of these drugs have limited effectiveness in controlling the progression of tumors [14] and the real clinical benefits of targeted therapy are inadequate [42]. Obstacles such as drug resistance, limited efficacy, relapse, and recrudescence hamper the development and clinical utilization of Ms in HCC [43]. Drawing lessons from combination chemotherapy regimens, the concept of co-inhibition of multiple targets or pathways has been proposed in an attempt to enhance the antitumor effect by simultaneously inhibiting multiple targets or pathways [44].

### Rationality of multitarget therapeutics

**Figure 1:**
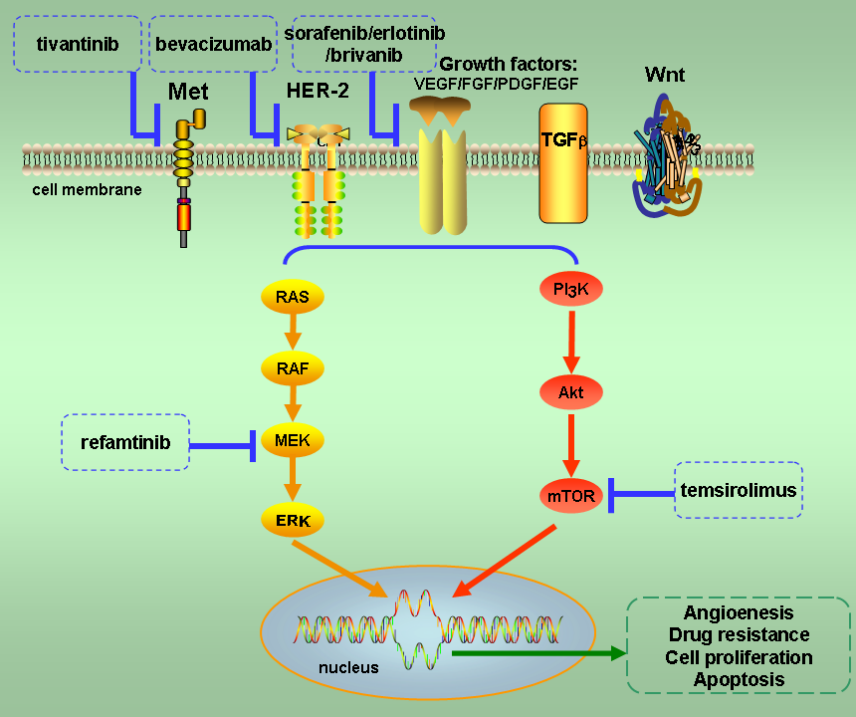
Major pathways of multiple target co-inhibition in advanced hepatocellular carcinoma Mutations in the RAS/RAF/MEK/ERK and PI3K/Akt/mTOR pathways enhance angiogenesis, drug resistance, cell proliferation, and apoptosis to facilitate the growth of cancer. These two pathways are the major targets of strategies involving co-inhibition of dual or multiple targets in the treatment of advanced HCC. The patterns of combined inhibition include dual targets at the level of growth factors and at the level of their downstream pathways. Molecular targeted agents involved in multiple target co-inhibition therapy are listed in this figure.

Numerous investigations concerning the mechanisms of resistance to MTAs have been conducted in recent years. Based on our current understanding, the mechanism of targeted therapeutic resistance is attributable to the complex systems of cell signal transduction and pathway networks [45]. The mainstream theory of targeted therapeutic resistance principally covers three aspects: (1) pathway redundancy, which is the ability of a signaling pathway to maintain an activated status even under inhibition by a targeted therapy [46]; (2) escape pathways, in which cell signal transduction can recruit an alternate signaling pathway through a cross-talk effect to escape attack on a targeted therapy [47]; (3) pathway reactivation, referring to the ability of a cell to reactivate the signaling pathway via downstream mutations despite inhibitory therapy [48]. In addition, heterogeneity is a considerable factor in tumor progression and drug resistance. Increasingly, studies have demonstrated the existence of abundant intra-tumor and inter-tumor heterogeneity [49–51]. Moreover, by applying the method of next-generation sequencing (NGS) polyclonality has been confirmed in multicentric HCC [52, 53], revealing independent tumorigenic occurrence. For these reasons, the strategy of suppressing only one target or pathway is no longer considered viable for treating HCC. Thus, to achieve greater efficacy in targeted therapy, inhibiting more than one target simultaneously may become a feasible approach to curb carcinogenesis as well as tumor progression. The concept of combining molecular target agents has been proposed and put into practice during the last few years [54], and several relevant clinical trials have been carried out for HCC. Here we categorize these trials as (1) combination blockade at the level of growth factor receptors (GFRs) and (2) combination blockade at the level of downstream pathways (Figure 1).

### Combination blockade at the level of GFRs

The balance between proangiogenic and antiangiogenic factors is crucial as carcinogenesis can be triggered when this balance is disturbed [55]. In addition, cancer cells, endothelial cells, and pericytes together change the microenvironment of the tumor [56]. Consequently, many GFRs, including EGFR, vascular endothelial growth factor receptor (VEGFR), platelet-derived growth factor receptor (PDGFR), c-mesenchymal-epithelial transition factor-1 (c-Met), and fibroblast growth factor receptor (FGFR), have been shown to be upregulated in HCC at the level of protein and gene expression [57]. Additionally, most of these GFRs belong to the family of RTKs. Because of the important role of RTKs in the initiation, progression, and maintenance of carcinomas, it is necessary to suppress RTKs adequately by therapy [58].

As mono-RTK targeted therapy shows low efficacy in the treatment of HCC, multitarget co-inhibition should be explored (Table 2). Although there is no evidence of crosstalk between EGFR and VEGF, a large percentage of clinical trials on HCC utilize EGFR-VEGF combination blockade for co-inhibition of these GFRs. This probably reflects the limited availability of approved clinical targeted drugs [59]. The outcomes in actual clinical practice varied among different research groups, so it is inappropriate to draw conclusions on the efficacy of this regimen of multitarget inhibition. For instance, Thomas et al. reported the outcome of a phase II clinical trial combining bevacizumab and erlotinib in patients with advanced HCC [60]. They studied 40 patients with advanced HCC and concluded that the combination of bevacizumab and erlotinib showed valuable antitumor activity in addition to being well tolerated. Another clinical trial conducted in Asian patients showed a similar outcome [61]. In this single-arm clinical trial for 51 participants who received erlotinib plus bevacizumab, the median PFS was 2.9 months and the median OS was 10.7 months; furthermore, grade 3/4 adverse events were infrequent and tolerable. These two trials demonstrate to a certain degree that use of bevacizumab plus erlotinib is effective for advanced HCC. Erlotinib, an EGFR inhibitor, has shown promising effects in alleviating fibrogenesis and controlling angiogenesis [62], whereas bevacizumab is an angiogenesis inhibitor. Although these two drugs each possess limited antitumor effects in advanced HCC, combined application shows encouraging outcomes.

As the most efficient agent used in monotherapy, sorafenib improves OS and PFS in patients with advanced HCC; however, its efficacy is limited and most patients ultimately die from the disease [63]. Therefore, researchers have investigated whether superior antitumor function could be accomplished by using sorafenib combined with other molecular agents targeting growth receptor factors. Many clinical explorations have been carried out using combinations such as brivanib plus sorafenib, erlotinib plus sorafenib, ramucirumab plus sorafenib, and regorafenib plus sorafenib (Table 2); however, the results of these clinical investigations are disappointing. For example, Zhu et al. showed no value from adding erlotinib to sorafenib in a comparison of the clinical benefit of sorafenib plus either erlotinib or placebo [64]. Llovet et al. evaluated 395 patients with advanced HCC who progressed during or after treatment or were resistant to sorafenib [65]. These patients were randomly assigned (2:1) to receive brivanib (a RTK inhibitor of VEGF and FGF) 800 mg orally daily plus best supportive care (BSC) or placebo plus BSC. The clinical benefits were similar between the two groups, indicating that sorafenib-based combination therapy showed no superiority over sorafenib monotherapy. Based on the outcomes of these clinical trials, it is not currently recommended to combine sorafenib with other targeted agents to treat advanced HCC.

**Table 2:** Clinical trials regarding dual or multiple targeted therapy (with published results)

Agents	Stage	Patients(n)	Therapeutic shceme	First or second line	Efficacy (combined therapy *vs* monotherapy)	Adverse events (AEs)	Ref
Erlotinib + Sorafenib	Phase 3	720	Sorafenib 400 mg bid po plus erlotinib 150 mg daily (*n* = 358) or sorafenib 400 mg bid po plus placebo 150 mg daily (*n* = 362)	First line	mOS: 9.5 *vs*. 8.5 months mTTP: 3.2 *vs*. 4.0 months ORR: 6.6% *vs* 3.9% DCR: 43.9% *vs* 52.5%	rash/desquamation, anorexia, diarrhea alopecia and HFSR.	[8]
Tivantinib + Sorafenib	Phase 1	20	Tivantinib: 240mg bid po plus sorafenib 400 mg bid po	Second line	ORR: 10% DCR: 65%	rash, diarrhea, and anorexia.	[9]
Brivanib + Sorafenib	Phase 3	395	Brivanib 800 mg po daily plus best supportive care (BSC) (*n* = 263) *vs* placebo plus BSC (*n* = 132).	Second line	mOS: 9.4 *vs*. 8.2 months mTTP: 4.2 *vs*. 2.7 months ORR: 10% *vs*. 2% DCR: 61% *vs* 40%.	hypertension, fatigue, hyponatremia, decreased appetite, asthenia, diarrhea, increased AST and ALT.	[10]
Bevacizumab + Erlotinib	Phase 2	51	Bevacizumab: 5 mg/kg i.v. plus erlotinib 150 mg po daily	First line	mPFS: 2.9 months mOS: 10.7 months.	rash, acne, diarrhea and gastrointestinal bleeding.	[11]
Bevacizumab + Erlotinib	Phase 2	40	Bevacizumab: 10 mg/kg i.v. plus erlotinib 150 mg po daily.	First line	mPFS: 9.0 months mOS: 15.7 months	fatigue, hypertension, diarrhea, elevated transaminases, gastrointestinal hemorrhage, wound infection thrombocytopenia.	[12]
Bevacizumab + Temsirolimus	Phase 2	28	Temsirolimus 25 mg i.v. plus bevacizumab 10mg/kg i.v.	First line	mPFS: 7 months mOS: 14 months ORR: 19%	cytopenias, fatigue, mucositis, diarrhea and mild bleeds.	[13]
Refametinib + Sorafenib	Phase 2	95	Refametinib 50 mg bid po plus sorafenib 200 mg (morning)/400 mg (evening) bid po	First line	DCR: 44.8% mTTP: 122 days mOS: 290 days	diarrhea, rash, aspartate aminotransferase elevation, vomiting and nausea.	[14]
Temsirolimus + Sorafenib	Phase 1	25	Temsirolimus 10 mg weekly po plus sorafenib 200 mg bid po.	First line	DCR: 68%	hypophosphatemia, infection, thrombocytopenia, HFSR and fatigue.	[15]

**Abbreviations:** AST: Aspartate Transaminase; ALT: Alanine Aminotransferase; HFSR: Hand-Foot Syndrome Reaction.

### Combination blockade at the level of the downstream pathway

Cell signal pathway reactivation, which is induced by mutation of downstream components, is the primary mechanism of resistance to MTAs [45]. In HCC, both the RAS/RAF/MEK/ERK pathway and the PI3K/Akt/mTOR pathway can be activated by gain-of-function mutations or overexpression of GFRs [66]. Moreover, either the RAS/RAF/MEK/ERK or PI3K/Akt/mTOR pathway has been frequently identified as the mutation hotspot of hepatocarcinogenesis in cases of resistance to targeted therapy (Figure 1) [67]. The increased antitumor efficiency of combination blockade at the level of the downstream pathway was confirmed in preclinical studies. Rudalska et al. used shRNA screening in a mouse model of HCC to identify Mapk14 (p38α) as one of the targets of sorafenib therapy resistance [68]. They demonstrated that elevated Mapk14-Atf2 signaling is a poor prognostic factor in sorafenib therapy of human HCC, which may translate to a promising novel approach to identify HCC patients with sorafenib resistance. In addition, the synthetic lethality of sorafenib plus a Mek inhibitor was verified *in vitro* using a medullary thyroid carcinoma cell line [69].

Based on these findings, Lim et al. reported the outcome of combination therapy with refametinib (a MEK inhibitor) plus sorafenib for Asian patients with advanced HCC [70]. In the 70 patients who received the study treatment, DCR was 44.8% and median TTP and OS were reached after 122 and 290 days, respectively. These outcomes seem to suggest that blockade upstream and downstream of a certain pathway both deserve further investigation. However, the toxicity produced by multitarget inhibition should be considered. Shimizu et al. performed retrospective research to evaluate the clinical effects and tolerability of a dual-targeting method involving the PI3K/AKT/mTOR and RAS/MEK/ERK pathways [71]. They reviewed 236 patients with advanced solid tumors who received treatments targeting the PI3K/AKT/mTOR and/or RAS/MEK/ERK pathways. Through deliberated assessment and comparisons they showed that although dual-pathway inhibition potentially showed promising efficacy compared with suppression of a single pathway, greater toxicity was the main obstacle to this therapeutic strategy. The rates of agent-related AEs greater than grade 3 were significantly higher in the dual-pathway group than with single-pathway inhibition (53.9% *vs*. 18.1%, *P* < 0.001). Given these results, for now clinicians are advised to be cautious with regard to combination blockade of multiple targets or pathways.

## TARGETED THERAPY COMBINED WITH TRANSCATHETER ARTERIAL CHEMOEMBOLIZATION (TACE)

Patients with advanced HCC with poor liver function and large tumor size have limited treatment options. TACE, a local treatment, is the first approach to provide a survival benefit in unresectable HCC [72]. TACE can be performed with diverse chemotherapeutic agents through infusion directly into the vessels supplying the tumor while blocking these vessels with a specific embolization material [73]. For a significant survival advantage, it is appropriate to combine TACE with targeted therapy in the treatment of advanced HCC [74].

It is reasonable to consider the combination of a local treatment (such as TACE) and antiangiogenic therapy (such as MTAs). First, increased levels of angiogenesis factors such as VEGF and angiopoietin can be triggered by the extensive ischemic necrosis caused by TACE [75]. Additionally, hypoxia induced by arterial embolization can promote the proliferation of tumor cells in addition to inhibiting apoptosis [76]. Hypoxia can also concurrently stimulate the production of several neo-angiogenesis factors including VEGF and IGF-2 [77]. Since angiogenesis plays a major role in tumor progression, as well as adaption and recurrence, a synergistic effect can be achieved when TACE is combined with targeted therapy aimed at upregulation of VEGF or other angiogenesis factors [78].

Several preclinical and clinical studies have shown that administration of multikinase inhibitors before or after TACE may target lesions distal to the TACE site, extend time to recurrence or progression, and improve survival (Table 3) [79]. Studies in laboratory models proved that the therapeutic efficacy of TACE was enhanced by antiangiogenic therapy through a recombinant adeno-associated virus vector encoding murine angiostatin [80]. In addition, a meta-analysis reported by Zhang et al. confirmed that OS, TTP, and overall response rate could be improved by combination therapy with TACE plus sorafenib in patients with intermediate or advanced HCC [81]. A worldwide prospective non-interventional study evaluating distinct patient subsets that compared the outcomes of sorafenib after TACE (*n* = 158) and sorafenib monotherapy (*n* = 29) confirmed that sorafenib can be used safely in combination with TACE [82]. Another attractive clinical trial, SPACE, assessed the efficacy of sorafenib plus TACE with doxorubicin-eluting beads (DEB-TACE) for intermediate-stage HCC. Its latest data revealed negative results for the improvement of TTP and clinical benefits in the combined therapy arm [83]. In this trial, 307 patients were randomly assigned to receive DEB-TACE plus sorafenib (*n* = 154) or placebo. The median OS was not reached, and TTP between each arm was similar, although DCR differed (89.2% for DEB-TACE plus sorafenib *vs*. 76.1% for DEB-TACE plus placebo). Interestingly, a longer median TTP was observed in the subgroup of Asian patients, which may be explained by the specific etiology of HCC. Additionally, brivanib, a dual inhibitor of VEGF and FGF signaling that suppresses the process of angiogenesis, was unable to improve OS when used as an adjuvant therapy to TACE [84].

Local treatment with TACE in patients with intermediate or advanced HCC in combination with anti-angiogenesis targeted therapy appears to be a feasible strategy. This approach has been demonstrated to be well tolerated; however, the efficacy of this type of combination should be further verified.

**Table 3:** Clinical trials regarding TACE combine with targeted therapy (with published results)

Agents	Stage	patients(n)	Therapeutic scheme	First or second line	Efficacy (combined therapy *vs* monotherapy)	Adverse events (AEs)	Ref
Sorafenib + TACE	Phase 3	458	Sorafenib 400 (*n* = 229) mg bid po or placebo (*n* = 227) po after 1-2 TACE	First line	mTTP: 5.4 *vs*. 3.7 months 3-months PFR: 65.0% *vs* 58.7% mOS: 29.7 months *vs*. NR	HFSR, elevated lipase, alopecia and rash/desquamation.	[16]
Sorafenib + TACE	Phase 2	304	Sorafenib 400 mg bid po (*n* = 82) + TACE *vs* TACE alone (*n* = 222)	First line	mTTP: 6.3 *vs* 4.3 months mOS: 7.5 *vs* 5.1 months DCR: 58.5% *vs* 44.5%	hand-foot skin reaction, alopecia and diarrhea, gastrointestinal bleeding, hyperbilirubinemia and hepatic encephalopathy.	[17]
Sorafenib + TACE	Phase 2	80	Sorafenib 400 (*n* = 31) mg bid po or placebo (*n* = 31) po after TACE	First line	mTTP: 9.2 *vs*. 4.9 months	anorexia, diarrhea, fatigue, hand–foot skin reaction, hematological event, nausea, rash/desquamation.	[18]
Sorafenib + TACE	Phase 2	43	Sorafenib: starting dose of 200 mg bid po increased to 400 mg bid in the majority of patients (*n* = 13), or placebo (*n* = 30) after TACE	First line	mOS: 20.6 *vs* 13.8 months;	pain, nausea, vomiting and mild elevation of liver enzymes.	[19]
Sorafenib + TACE	Phase 2	355	Sorafenib 400mg bid po plus TACE (*n* = 164) *vs* sorafenib alone (*n* = 191)	First line	mTTP: 2.5 *vs* 2.1 months; mOS: 8.9 *vs* 5.9 months.	hand-foot skin reaction	[20]
Sorafenib + TACE	Phase 2	45	Sorafenib 200mg bid po plus TACE	First line	mOS: 27 *vs*. 17 months	hand-foot skin reaction, rash and diarrhea.	[21]
Bevacizumab + TACE	Phase 2	32	Bevacizumab (5 mg/kg) (*n* = 16) or placebo (*n* = 16)	First line	mTTP: 7.2 *vs*. 11.7 months mOS: 5.3 *vs* 13.7 months	severe bleeding, vascular, and septic events; right heart dilatation, anorexia, fatigue, or alopecia were low-grade events	[22]
Bevacizumab + TACE	Phase 2	30	Bevacizumab 10 mg/kg IV (*n* = 15) or placebo (*n* = 15).	First line	mOS: 49 *vs*. 61 months 16-weeks PFS rate: 79% *vs* 19%.	elevated transaminases, pain, pyrexia, nausea/vomiting, and fatigue.	[23]
Sunitinib + TACE	Phase 2	103	Sunitinib (*n* = 38) 37.5 mg po daily or placebo (*n* = 65) after TACE	First line	mOS: 8.8 *vs* 6.3 months mTTP: 3.9 *vs* 2.5 months	hrombocytopenia, fatigue, leukopenia, and anemia. upper gastrointestinal bleeding, hepatic encephalopathy, and hyperbilirubinemia.	[24]
Sunitinib + TACE	Phase 2	16	Sunitinib 37.5 mg po daily after TACE	First line	mPFS: 8 months mOS: 14.9 months DCR: 81%.	thrombocytopenia, increase of amylase/lipase, lymphopenia, and fatigue.	[25]

## TARGETED THERAPY COMBINED WITH OTHER TREATMENTS

Other treatments for advanced HCC, such as radiotherapy, PEI, immunotherapy, and even surgical resection, have also been attempted in combination with targeted therapy. Some combinations have shown promising outcomes. For example, enhanced radiosensitivity could be achieved through combined targeted inhibition of Sonic Hedgehog (SHH) [85]. Combination therapy with sorafenib and radiofrequency ablation (RFA) was associated with a better OS and a lower incidence of post-RFA recurrence compared with RFA alone (Table 4). However, this has only been confirmed in a small-sample randomized controlled trial [86] and in retrospective studies [87].

For patients who obtain apparent tumor remission during ongoing targeted therapy, the options for further therapy should be evaluated. Curtit et al. reported a case in which a potential benefit was gained from pre-surgery targeted therapy. A 56-year-old man with advanced HCC in the context of a long history of hepatitis C-related cirrhosis showed obvious tumor suppression after 6 months of sorafenib treatment. The size of the liver tumor was reduced, providing the opportunity to perform tumor resection. Pathologic examination indicated that complete histologic response was achieved [88]. It is worth verifying whether targeted therapy could be used as an adjuvant treatment before surgical excision or radiotherapy in advanced HCC.

Although the outcomes of single MTAs applied to date in the treatment of advanced HCC are depressing, improved therapeutic effect might be attained for novel targeted agents such as p53 mutation, which is one of the most frequent driver mutations in patients with HCC [89]. Gene therapy targeting p53 in liver cancer revealed encouraging activity in inhibiting carcinogenesis although MTAs that inhibit p53 are currently not available [90, 91]. Li [90] demonstrated a synergetic increase in the therapeutic efficacy of trans-arterial embolization (TAE) and targeted gene therapy through application of a polyplex formed by surface modified nHAP and p53-expressing plasmid as a gene vector in hepatoma-targeted TAE gene therapy. Moreover, targeted overexpression of tBid or BikDD has been validated as a method to reduce tumor growth [92–94], especially when combined with chemotherapy [95, 96]. Bik is a BH3-only protein of the Bcl-2 family that promotes apoptosis and tBid (p15) is one of the fragments of Bid released in response to apoptotic stimuli. BikDD is a Bik mutant containing changes in T33D and S35D that mimic phosphorylation at these two residues, thus enhancing binding affinity with the antiapoptotic proteins Bcl-XL and Bcl-2 [97]. Mono or combined treatment including gene therapy targeting vital genes such as p53, TERT, and Bik, is a promising new direction of study.

In addition, because immunotherapy such as “immune checkpoint” inhibition has been demonstrated to be a novel and effective treatment for solid tumors, it will also be valuable to confirm whether enhanced antitumor efficacy could be achieved through targeted therapy combined with immunotherapy.

**Table 4:** Other combination regimens based on targeted therapy in hepatocellular carcinoma (with published results)

Agents	Stage	Patients(n)	Therapeutic scheme	First or second line	Efficacy (combined therapy *vs* monotherapy)	Adverse events (AEs)	Ref
Radiofrequency ablation (RFA) + Sorafenib	Phase 2	128	Radiofrequency ablation plus sorafenib (400mg bid) (*n* = 64) *vs* radiofrequency ablation alone (*n* = 64)	Both	mOS: 161.8 *vs* 118.6 weeks. The 1-, 2- and 3- year cumulative incidences: 62.8%, 85.4% and 92.7% *vs* 40.5%, 62.9% and 74.5%.	gastrointestinal bleeding, pleural effusion requiring drainage, mild or moderate increase in body temperature.	[87]
Radiofrequency ablation (RFA) + Sorafenib	Phase 2	45	Radiofrequency ablation plus sorafenib (400mg bid) (*n* = 15) *vs* radiofrequency ablation alone (*n* = 30)	Both	RFA-induced ablated area: 46.3 mm ± 10.3 and 33.0 mm ± 6.9 *vs* 32.9 mm ± 7.6 and 25.6 mm ± 5.7.	serum asparatate aminotransferase concentration transient increases, handfoot skin reaction.	[116]
Radiofrequency ablation (RFA) + Sorafenib	Phase 2	62	Radiofrequency ablation plus sorafenib (400mg bid) (*n* = 30) *vs* radiofrequency ablation alone (*n* = 32)	First line	recurrent rate: 56.7% *vs* 87.5%; mTTP: 17.0 *vs* 6.1 months;	hand-foot skin reactions, diarrhea, fatigue, alopecia and hypertension.	[86]

## PERSPECTIVE AND FUTURE DIRECTION

**Figure 2:**
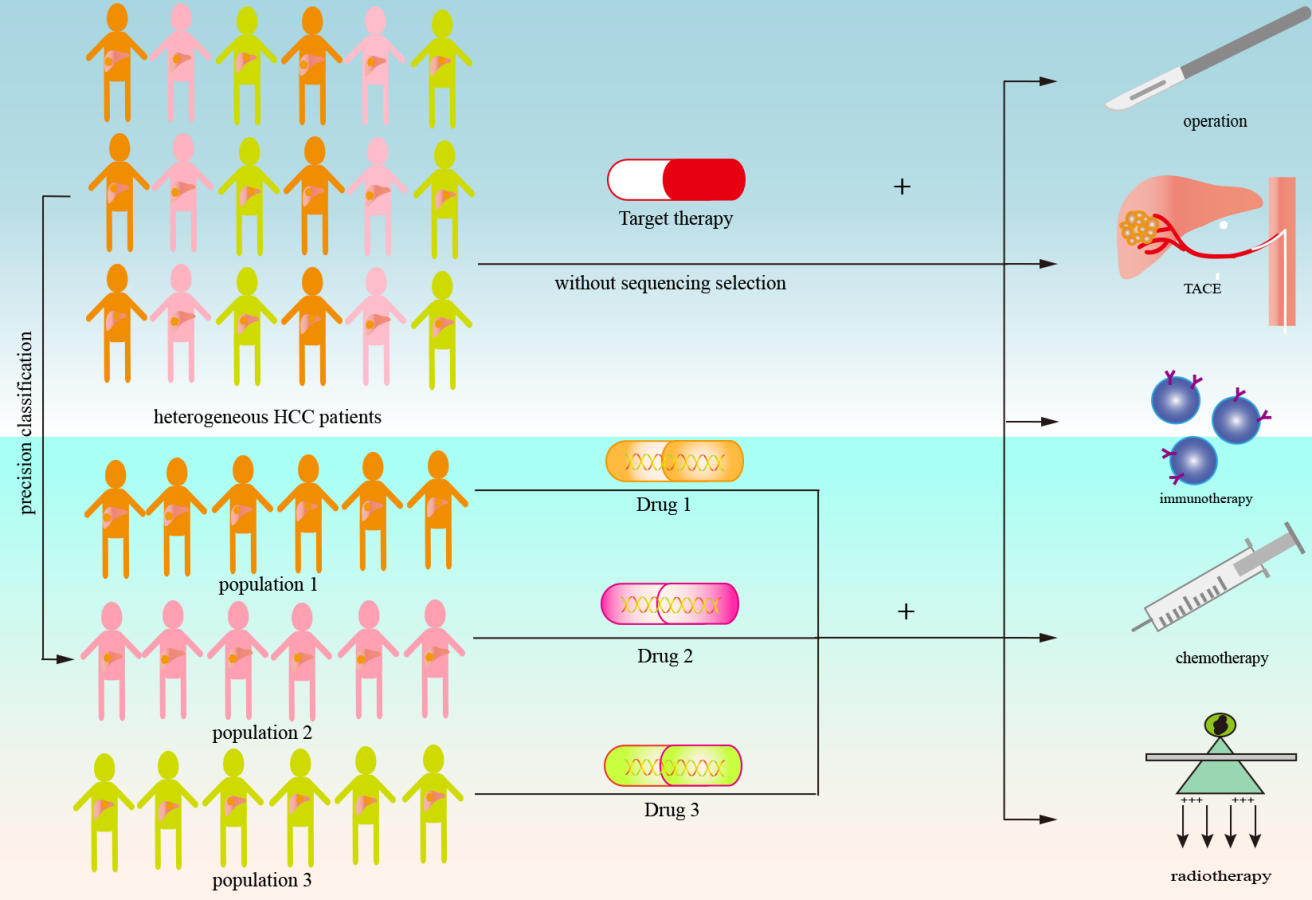
Schematic of combination treatment based on targeted therapy in advanced hepatocellular carcinoma In patients with advanced HCC, combination treatment based on targeted therapy involves molecular targeted agents combined with other modalities such as surgery, TACE, immunotherapy, chemotherapy, or radiotherapy. The lack of precise target population selection may be the primary reason for limited cancer control using these strategies. Treatment will become more precise and effective through effective screening of patients with potential benefits. This may be achieved by genome sequencing to identify therapeutic targets or by more reliable molecular classification of the tumor.

The concept of MTA combination therapy is still in its infancy and several problems have arisen in these pioneer studies. On one hand, the schema of most clinical studies is based on a “trial and error” model [98]. For the obscure landscape of cell signal networks and tumor escape mechanisms, the therapeutic combination approaches selected to date have largely been decided based on the empirical work of clinicians. On the other hand, it is hard to judge whether combination therapy is superior to targeted therapy alone because of the lack of an MTA control arm in most clinical trials. Besides, the criteria for eligible patients in these trials almost always exclude patients with specific genomic mutations or particular biomarkers, which may be the primary reason for the negative results in comparisons of MTA combination therapy. Combination therapy will become more precise and effective through screening of patients with potential benefits (Figure 2).

Deeper understanding of the mechanisms of tumor occurrence, progression, development, and metastasis will facilitate rational development and accelerate the adoption of MTA combination therapy in HCC. To confront the challenges of MTA combination therapy, a multi-disciplinary team approach, including precision medicine, may be desirable [99]. With the development of next-generation sequencing technology, the genomic profiles of patients with advanced HCC can now be obtained [100]. This may allow us to identify valuable mutations that could represent inhibition targets and guide clinicians in deciding which targets should be combined. More significantly, more potent antitumor effects might be observed by selecting the most appropriate patients to receive targeted combination therapy [43]. Most importantly, combining biopsy/rebiopsy and precise genome-wide sequencing can provide credible information and evidence concerning the tumor response to the targeted therapy [101]. Therefore, the importance of biopsy/rebiopsy in accurately identifying potential patients who will benefit from MTA combination therapy should be emphasized.

In conclusion, numerous studies have applied combination treatments based on targeted therapies in HCC, including targeted therapy plus chemotherapy, dual or multiple targeted therapy, targeted therapy plus radiotherapy, and targeted therapy plus TACE. Although some of these explorations yielded informative and promising results, the majority showed negative or even inferior outcomes. So far, there have been few triumphs with the application of combination therapy in the clinic despite encouraging results from *in vitro* studies. We do not yet have an effective therapeutic approach to control and cure advanced HCC in humans, and there is a long road ahead on the journey toward optimal tumor treatment.
